# Thermoresponsive Poly(*N*-Isopropylacrylamide-*co*-Dimethylaminoethyl Methacrylate) Microgel Aqueous Dispersions with Potential Antimicrobial Properties

**DOI:** 10.3390/polym11040606

**Published:** 2019-04-02

**Authors:** Coro Echeverría, Alejandro Aragón-Gutiérrez, Marta Fernández-García, Alexandra Muñoz-Bonilla, Daniel López

**Affiliations:** 1 Instituto de Ciencia y Tecnología de Polímeros (ICTP-CSIC), C/Juan de la Cierva 3, 28006 Madrid, Spain; alejandro.aragon.gutierrez@gmail.com (A.A.-G.); martafg@ictp.csic.es (M.F.-G.); sbonilla@ictp.csic.es (A.M.-B.); 2 Interdisciplinary Platform for Sustainable Plastics towards a Circular Economy-Spanish National Research Council (SusPlast-CSIC), 28006 Madrid, Spain

**Keywords:** polycationic microgels, quaternization, antimicrobial activity, temperature-responsive, fractal analysis

## Abstract

The work herein describes the preparation of thermoresponsive microgels with potential antimicrobial properties. Most of the work performed so far regarding microgels with antimicrobial activity, deals with the ability of microgels to carry and release antibiotics or antimicrobial agents (antimicrobial peptides). The originality of this work lies in the possibility of developing intrinsic antimicrobial microgels by copolymerization of the well-known thermoresponsive monomer, *N*-isopropylacrylamide (NIPAM) with dimethylaminoethyl methacrylate (DMAEMA), a water-soluble monomer, to form microgels via precipitation polymerization (radical polymerization). Due to the presence of a tertiary amine in the DMAEMA comonomer, microgels can be modified by *N*-alkylation reaction with methyl and butyl iodide. This quaternization confers positive charges to the microgel surfaces and thus the potential antimicrobial activity. The effect of DMAEMA content and its quaternization with both, methyl and butyl iodide is evaluated in terms of thermal and surface charge properties, as well as in the microgel size and viscoelastic behavior. Finally, a preliminary study of the antimicrobial activity against different microorganisms is also performed in terms of minimum inhibitory concentration (MIC). From this study we determined that in contrast with butylated microgels, methylated ones show potential antimicrobial activity and good physical properties besides of maintaining microgel thermo-responsiveness.

## 1. Introduction

Microgels, intramolecularly cross-linked submicron size polymer particles are an interesting subset of polymer gels that can be designed to respond to environmental external stimuli like temperature, magnetic field, pH, ionic strength or electric field [1,2]. After decades of research, polymeric microgels have demonstrated to be versatile systems with potential applications as diverse as industrial applications as paints, ink jets printing, separation [3] and biomedical applications such as drug delivery systems [4,5], biomimicking artificial synovial fluids [6], tissue mimicking [7] and injectable 3D cell scaffolds [8]. Besides the mentioned advanced applications, microgel can be used as building blocks to create structures such as colloidal crystals, films and gels in the macroscopic scale [9,10,11,12], but also as active sites incorporated within functional polymer matrices so that the design of tailored multifunctional materials can be obtained [13,14]. Following this idea, in a recent work we used thermoresponsive poly(*N*-isopropylacrylamide), (PNIPAM) based microgels as active sites encapsulated into polymeric nanofiber as a first approach toward the design of new active wound dressings [15,16]. As a continuation, in the present work we aim for the development of intrinsic antimicrobial stimuli-responsive microgels that could be used as active site so that antimicrobial activity could be imparted.

The development and use of antimicrobial materials have become necessary to overcome the increasing problem of infectious diseases and multi-resistant microorganisms, the latter being one of the greatest threats for health and health care systems as stated by the World Health Organization [17]. Polymeric materials with antimicrobial activity have been widely studied [18] and pointed out as suitable candidates to overcome this problem. Most of the polymers with intrinsic antimicrobial activity are positively charged. In fact, among the different types of biocide polymers used, those with quaternary ammonium groups are probably the most explored ones. This is thanks to their properties such as chemical stability and their difficulty to permeate through the skin of human or animals [18,19]. Since most bacterial cell walls are negatively charged, it is generally accepted that polycationic polymers interact electrostatically with the negative charged bacteria cell wall, causing disruption of the membrane and posterior death [20,21].

Most of the work done regarding microgels with antimicrobial activity deals with the drug delivery ability of microgels capable to carry and release antibiotics or antimicrobial agents [22,23,24,25]. Malmsten et al. first studied the existing interactions between antimicrobial peptides (AMP) and poly(acrylic acid) microgels and peptide uptake and sustained release [26,27,28]. In a more recent study, they also studied key factors for the encapsulation and further release of AMP from poly(ethyl acrylate-*co*-methacrylic acid) anionic microgels by analyzing the effect of microgels different charge density in the AMP uptake/release [29]. As a continuation, authors took advantages of the developed anionic microgels, and designed a covalently bounded microgel coating, where AMP was further loaded. Here again, uptake and release of AMP was studied and the obtained surface tested against different microorganisms, proving its antimicrobial activity with AMP controlled release [24]. Stular et al. followed a different strategy to impart antimicrobial activity to microgel systems. In this case, they encapsulated silver nanoparticles into PNIPAM-Chitosan based temperature- and pH-responsive microgel systems; with the purpose of further release of the nanoparticles. Authors applied this system to model cotton fabric and suggested that this strategy would contribute to the development of smart antimicrobial textiles. However, the above-mentioned strategies required the release of the antimicrobial agent (AMP, silver nanoparticles), and therefore, the antimicrobial effect is not permanent.

Our approach consists in the copolymerization of NIPAM with dimethylaminoethyl methacrylate (DMAEMA), a water-soluble monomer containing a tertiary amine group, which confers some additional properties to PNIPAM microgels, such as pH sensitivity [30,31]. In addition, these tertiary amine groups are susceptible of quaternization to give positive charge, thus providing antimicrobial character to the hydrogel. To our knowledge this is the first attempt in which intrinsic antimicrobial microgels are developed. In this work, we also report the effect of DMAEMA content and its quaternization (via alkylation reaction with different lengths of the quaternizing agents, methyl iodide and butyl iodide), on the hydrodynamic diameter, surface charge, thermal properties of the obtained microgels as well as the influence in the viscoelastic properties of microgel dispersion. To determine the viscoelastic properties and how microgel particles might interact and assemble (or not) under deformation, it is crucial for the further confinement of microgels in the development of multifunctional active material (dressing) which is the ultimate objective of this on-going research.

## 2. Materials and Methods

### 2.1. Materials

For the synthesis of the microgels: *N*-isopropylacrylamide (NIPAM, 99%) and 2-(dimethylamino)ethyl methacrylate (DMAEMA, 98%, Aldrich, St. Quentin Fallavier, France) were used as monomers. *N*,*N*’-Methylenbis(acrylamide) (MBA, Aldrich), was used as the crosslinking agent and ammonium persulfate (APS, Aldrich) was the initiator of the reaction. Iodomethane (Aldrich, 99%) and 1-iodobutane (Aldrich, 99%) were employed as the alkylating agent. Anhydrous dimethylformamide (DMF) (99.5% purity) and hexane (95%) was supplied by Sigma Aldrich, as used as received.

For the microbiological assays: Sodium chloride (NaCl, 0.9%, BioXtra, Steinheim, Germany, suitable for cell cultures) and phosphate buffered saline (PBS, pH 7.4) were purchased from Aldrich. The microbial growth media, BBL^TM^ Mueller Hinton broth was obtained from Becton, Dickinson and Company (Madrid, Spain). Sheep blood (5%) Columbia Agar plates were acquired from BioMérieux (Madrid, Spain). Gram-positive *Staphylococcus aureus* (*S. aureus*, ATCC 29213) and *Staphylococcus epidermidis* (*S. epidermidis*, ATCC 12228), were used as bacterial strains, and the yeast *Candida parapsilosis* (*C. parapsilosis*, ATCC 22109) was used as fungal strain and purchased from Oxoid (Wesel, Germany).

### 2.2. Synthesis of Microgels

**Scheme 1:**
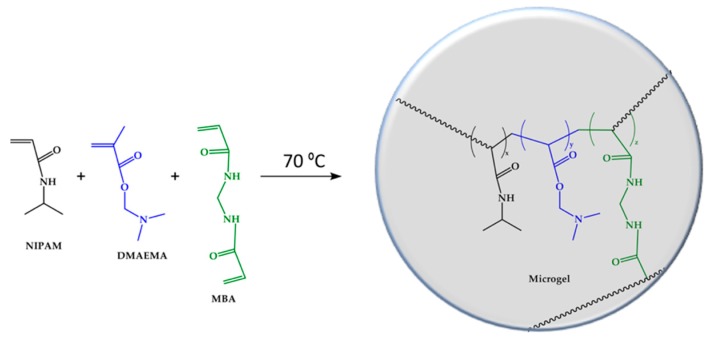
Polymerization reaction.

Surfactant-free emulsion polymerization was used for the synthesis of poly(*N*-isopropylacrylamide-*co*-dimethylaminoethyl methacrylate) (P(NIPAM-*co*-DMAEMA)) microgels. Four different compositions of P(NIPAM-*co*-DMAEMA) microgels were prepared with respect to DMAEMA monomer, NIPAM:DMAEMA ratios of 100:0, 85:15, 80:20 and 75:25 wt.%. Polymerization reactions were carried out in a 250 mL round-bottomed four-necked flask equipped with a condenser, argon purge line and overhead mechanical stirrer. The weight percentage of crosslinker (MBA) and initiator (APS) was 15% and 10%, respectively. NIPAM, DMAEMA and MBA were added to a total volume of 200 mL distilled water. This mixture was then purged with argon gas for 30 min to remove oxygen and homogenized at 500 rpm for 10 min. After homogenization, the polymerization was thermally initiated by immersion of the reaction flask in a 70 °C bath and the immediate addition of the APS [30], which was previously dissolved in 2 mL of distilled water. After adding the initiator, the mixture became immediately turbid and the polymerization reaction proceeded under argon atmosphere, stirred at 450 rpm, for 8 h. The microgel dispersion was allowed to cool down to room temperature and then, was dialyzed (molecular weight cutoff = 3500 Da) against distilled water to remove any unreacted monomer and other impurities. The washed polymer material was then frozen overnight and lyophilized. (Polymerization reaction is shown in Scheme 1).

### 2.3. Quaternization of Microgels

P(NIPAM-*co*-DMAEMA) microgels were quaternized using two different alkylating agents: (i) Methyl iodide, and (ii) butyl iodide. The synthetic methodology for the quaternization was as follows: A dispersion of microgels (200 mg) in 5 mL anhydrous DMF was introduced in an appropriately sized sealed tube containing a magnetic stirrer bar. Then, a large excess of alkyl agent (molar ratio copolymer:methyl iodide ≈ 1:4) was added. The mixture was purged with argon for 20 min, stirred and heated at 70 °C for 12 days to ensure the complete reaction [21]. After that period, the mixture was cooled down to room temperature and was poured into a vigorous stirring solution of n-hexane, producing a phase separation. The polar phase was diluted with DMF and H_2_O and purified by dialysis (molecular weight cutoff = 3500 Da) against distilled water to remove residual products.

### 2.4. Characterization Methods

FTIR measurements of lyophilized microgels were carried out at room temperature in attenuated total reflection (ATR) mode with a spectrometer (Perkin–Elmer Spectrum Two). A background spectrum was obtained before each test to compensate by spectral subtraction the presence of CO_2_ or the humidity effect. Spectra were obtained in the 4000–500 cm^−1^ region.

Dynamic Scanning Calorimetry (DSC) was used to determine the glass transition temperature (T_g_) of the PNIPAM, P(NIPAM-*co*-DMAEMA) non-quaternized and quaternized microgels (P(NIPAM-*co*-DMAEMA)-Q). Experiments were carried out using a DSC calorimeter Q-2000 provided by TA Instruments. The thermograms were performed under nitrogen atmosphere with a flow of 50 mL min^−1^ and heating and cooling rates for the runs were 10 °C min^−1^. The typical sample weight was around 3.5 mg and was weighted in an electronic autobalance Perkin–Elmer AD4. The cycle program consisted of a first heating stage from −20 °C to 220 °C at a rate of 10 °C min^−1^ followed by cooling to −20 °C and subsequent heating up to 250 °C at the same rate. The glass transition temperature was obtained from the second heating. The midpoint of the inflection was taken as T_g_.

^1^H NMR was used to calculate the degree of quaternization of P(NIPAM-*co*-DMAEMA)-Q microgels. Spectra were obtained using a Bruker 400 MHz spectrometer (Avance III HD-400) at room temperature with D_2_O as solvent.

For the analysis of the Zeta potential and hydrodynamic diameter (Dh), a Zetasizer Nano series ZS Dynamic Light Scattering (DLS) (Malvern. Instruments Ltd., UK) was employed. The hydrodynamic diameters of the microgels were determined at 20 °C (swollen state) and 40 °C (collapsed state). For the Zeta Potential, measurements were carried out at 20 °C but also at 37 °C (and at neutral pH) in order to simulate the antimicrobial test conditions.

Rheological tests were carried out on an ARG2 rheometer (TA Instruments). Parallel acrylic plate geometry with a diameter of 60 mm was used. Microgels were dispersed in distilled water at the following concentrations: 1, 2 and 5 wt% (g mL^−1^). Strain sweep measurements were performed between 0.01 and 100% strain at a constant and nondestructive frequency of 0.5 Hz and at a temperature of 20 °C. A minimum of five measurements of each sample were carried out in order to observe the reproducibility and obtain an average of the determined parameters.

### 2.5. Evaluation of the Antimicrobial Properties

Antimicrobial activities of the microgels were evaluated by determining their minimum inhibitory concentrations (MIC) against the bacterial strains *S. aureus* and *S. epidermidis*, and the fungal strain *C. parapsilosis* by standard broth microdilution method. Each copolymer was first dispersed in water and then diluted with sterile Mueller–Hinton (MH) broth to obtain a final concentration of 20 mg mL^−1^. Bacterial and fungal strains were cultured in 5% sheep blood Columbia agar plates for 24 h in an incubator at 37 °C, then dispersed and adjusted with saline solution to a turbidity equivalent to 0.5–1 McFarland standard (1–3 × 10^8^ colony forming units (CFU) mL^−1^) [32]. This suspension was further diluted (1:100) with Mueller–Hinton broth to yield the suspension working solution (10^6^ CFU mL^−1^). As general broth microdilution procedure, 100 µL from each one of polymer dispersions were placed in the first column of a 96-well round-bottom microplate. The rest of the columns were filled with 50 µL of broth and from the first columns, 50 µL were transferred to the next well and subsequent 1:2 serial dilutions were made across the plate. Finally, 50 µL of the corresponding microbial working dispersion was added to each well. A well without antimicrobial polymer, containing only the tested microorganism, was used as positive growth control [33]. After incubation at 37 °C for 24 or 48 h for bacteria and fungi, respectively, the MIC was visually estimated from the lowest concentration of copolymer at which the growth of microorganisms was inhibited. The starting microgel concentration was 20 mg mL^−1^.

## 3. Results and Discussion

### 3.1. P(NIPAM-co-DMAEMA) Microgels Preparation and Quaternization

**Figure 1:**
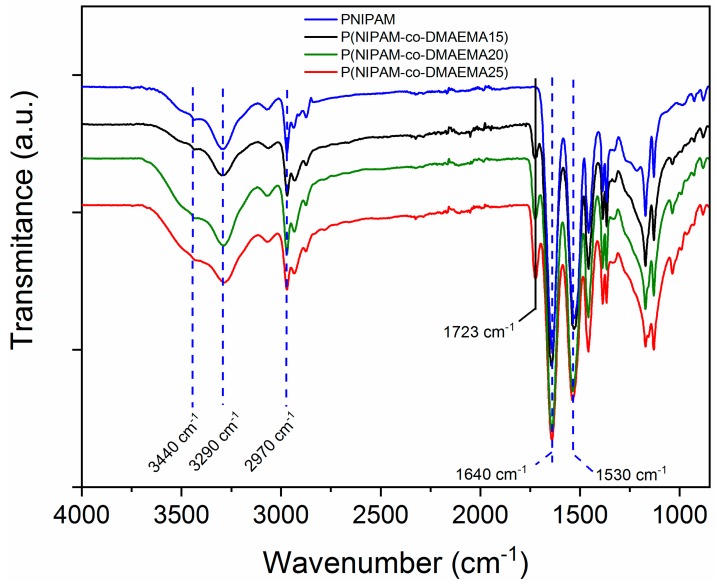
FTIR/attenuated total reflection (ATR) spectra of the poly(*N*-isopropylacrylamide-*co*- dimethylaminoethyl methacrylate), P(NIPAM-*co*-DMAEMA) microgels.

In order to confirm that the copolymerization of NIPAM and DMAEMA was successfully achieved we analyzed the obtained structure by FTIR/ATR. Figure 1 shows the spectra of P(NIPAM-*co*-DMAEMA) microgels in the 4000–500 cm^−1^ region. For the sake of clarity, the microgels are labeled as PNIPAM, P(NIPAM-*co*-DMAEMA15), P(NIPAM-*co*-DMAEMA20) and P(NIPAM-*co*-DMAEMA25) for those of NIPAM:DMAEMA ratios of 100:0, 85:15, 80:20 and 75:25 wt.%, respectively. The main FTIR assignments in PNIPAM are the absorption bands which appeared at 3290 cm^−1^ and 3440 cm^−1^ (secondary amide N–H stretching), 2970 cm^−1^ (–CH_3_ asymmetric stretching), 1640 cm^−1^ (secondary amide C=O stretching, amide I bond), and 1530 cm^−1^ (secondary amide C=O stretching, amide II bond) [34]. In the spectra of microgels with DMAEMA, the addition of this comonomer leads to the emergence of the characteristic band assigned to ester C=O group at 1723 cm^−1^, which confirms the successful copolymerization of PNIPAM with DMAEMA [35]. In addition, this band increases in intensity as the content of DMAEMA augments in the microgel.

Since the main objective of the work was the development of polycationic microgels that might present antimicrobial activity, the next step of the work consisted in the quaternization of the tertiary amine of DMAEMA by the *N-alkylation* reaction with methyl iodide and butyl iodide following the procedure described in the experimental section. The degree of quaternization was calculated by comparing 1H NMR spectra of the hydrogel before and after the quaternization reaction with methyl and butyl iodide (see Figure S1 in the Supporting Information). It is clearly observed that the peak assigned to the dimethyl group showed a different chemical shift in the quaternized analogues. Initially, it appeared at 2.85 ppm and after quaternization it shifted at a higher displacement, δ = 3.18 ppm. The fact that the signal at 2.85 ppm practically disappears in P(NIPAM-*co*-DMAEMA25-QMe) quaternized microgels spectra, indicates the almost quantitative reaction of nitrogen atoms. However, in the case of P(NIPAM-*co*-DMAEMA-QBu), it was calculated a degree of quaternization of about 70% probably due to the steric hindrance effect.

### 3.2. Effect of DMAEMA Content and Its Quaternization in the Thermal Properties of Microgels

**Figure 2:**
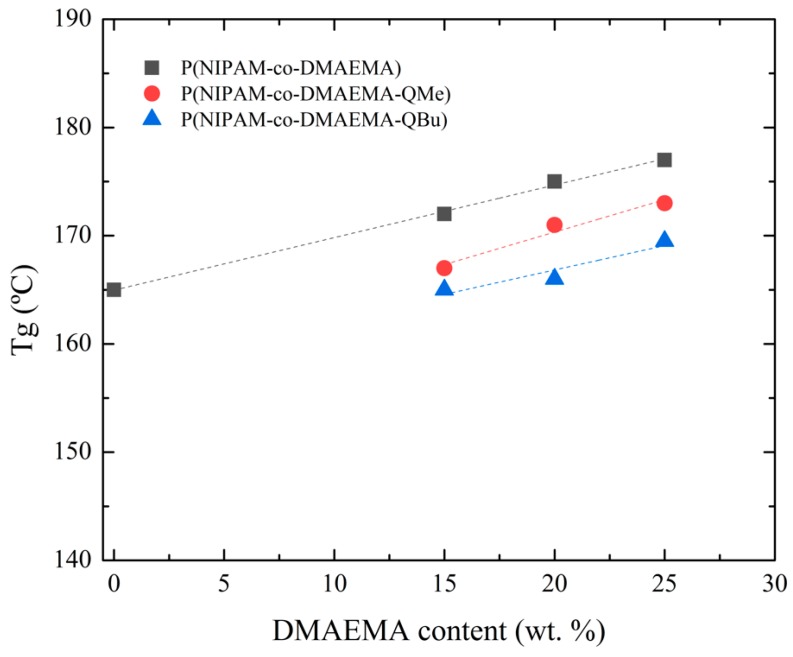
Effect of DMAEMA and its quaternization in the T_g_ of the final microgels.

Figure 2 reports the evolution of the glass transition temperature of the samples as a function of the DMAEMA content for unquaternized, methyl quaternized (QMe) and butyl quaternized (QBu) microgel samples. (T_g_ values were obtained from DSC thermograms collected in Figure S2 of the Supporting Information.) The glass transition temperature obtained for PNIPAM microgels is approximately 165 °C. This value is higher than the one reported in the literature [36]. This increase can be explained as a result of crosslinking, which reduces significantly the mobility of polymer chains; that is, a higher temperature is needed to promote some mobility, characterizing the glass transition [36]. For unquaternized microgels, single glass transition temperatures were noticed, which indicates the formation of miscible/homogeneous copolymers.

By analyzing the results shown in Figure 2, it was observed that the T_g_ of unmodified microgels slightly increased when increasing the DMAEMA content. However, the glass transition temperature reported in literature of pure PDMAEMA is 18.5 °C [37], and the copolymerization of PNIPAM with a flexible comonomer such as DMAEMA, should produce a decrease in T_g_ values with the addition of DMAEMA. As observed in Figure 2, the T_g_ values not only did not decrease, but they slightly increased with the incorporation of DMAEMA content. This result suggests some kind of interaction (hydrogen bond formation) between NIPAM and DMAEMA. Additionally, since the MBA concentration was kept constant, the increase in the glass transition temperature can be also understood as an improvement in the crosslinking distribution inside the microgel network by the incorporation of DMAEMA comonomer derived from the aforementioned interactions. As for the quaternization effect, although the trend followed with the increase of DMAEMA is the same, T_g_ values were smaller in comparison with unquaternized microgels. This could be due to a change in the interactions and, thus, the breakage of the suggested hydrogen bond formation between NIPAM and DMAEMA units, which may help the polymer chain mobility.

### 3.3. Effect of DMAEMA in the Viscoelastic Properties of Microgel Dispersions: Fractal Analysis

**Figure 3:**
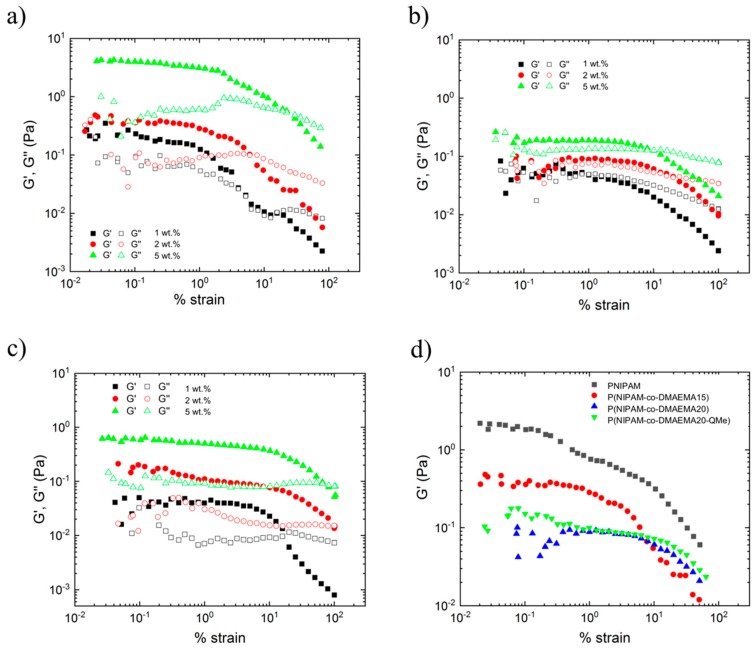
Representative curves of the elastic (G’) and viscous (G’’) modulus as a function of % strain for the samples: (**a**) P(NIPAM-*co*-DMAEMA15), (**b**) P(NIPAM-*co*-DMAEMA20) and (**c**) quaternized P(NIPAM-*co*-DMAEMA20-QMe) (Methyl iodide, QMe), measured at three different concentrations of microgel dispersion: 1, 2 and 5 wt.% (**d**) Comparison of the G’, G’’ evolution with % strain for PNIPAM, P(NIPAM-*co*-DMAEMA15), P(NIPAM-*co*-DMAEMA20) and quaternized P(NIPAM-*co*-DMAEMA 20-QMe) samples with 2 wt.% microgel concentration.

Figure 3a shows the evolution of the elastic modulus, G’, and the viscous modulus, G’’, with the % strain in P(NIPAM-*co*-DMAEMA15) microgels at different concentrations: 1, 2 and 5 wt.% (g mL^−1^). At low strain rates, G’ and G’’ present constant values with more elastic than viscous character (G’ > G’’). This zone determines the linear viscoelastic region (LVR), where the elastic and the viscous modulus were independent from the applied strain, until reaching a critical deformation, γ_0_, where G’ and G’’ started dropping. At higher strain rates, it is seen that G’ and G’’ crossover. From this point, the viscous character of the samples is higher than the elastic, G’’ > G’. With respect to the influence of the microgels concentration, it can be observed G’ and G’’ increased when increasing the concentration; that is, the elastic character is increased by increasing microgels content. Similar behavior was observed for the PNIPAM microgel sample containing 20% of DMAEMA (Figure 3b) and for the methyl quaternized P(NIPAM-*co*-DMAEMA20) microgel sample (see Figure 3c).

Regarding the effect of DMAEMA content and quaternization, in Figure 3d the evolution of the elastic modulus, G’, with % strain in microgels at a concentration of 2 wt.% (g mL) is shown. On the one hand, it can be appreciated that the plateau modulus, G’_0_, decreased by increasing DMAEMA content. On the other hand, it could be determined that the critical deformation, γ_0_, increased by increasing DMAEMA concentration. No differences were observed when comparing unquaternized and quaternized P(NIPAM-*co*-DMAEMA20) microgel system.

The fact that microgel dispersion shows a solid-like behavior (G’ > G’’) in the LVR could be indicative of a more complex structure formed from interactions occurring between microgels, as it was already described in previous works [38,39,40,41]. Such structures break upon deformation above certain % strain (critical strain). Besides, it is worth mentioning the slight increases (maximum) detected for G’’ at high strains (at the beginning of the non-linear range). This behavior is known as a weak strain-hardening, phenomenon that is directly associated to dissipated energy. In this case, this energy dissipation could come from the breakage of the complex structures that microgel dispersion might have formed [42].

In the light of the obtained results, we could consider that microgel particles are indeed interacting and probably developing cluster or agglomerates, as it has already observed in previous studies [39,40,41]. To elucidate the structure formation of the microgel dispersion from rheological measurements and correlate it with their elastic properties, we performed a fractal analysis based in two fractal models: Shih et al. [43] and Wu and Morbidelli [44] models. Both models consider that the structure of the colloidal gel is like a collection of aggregates or flocs: Fractal objects with average size and fractal dimension. As established in the literature, microgels can also be considered as colloidal gels, showing a highly disordered structure that at certain length scales are often self-similar. Then, microgels can be described in terms of fractal geometry.

In solid-like colloidal gels the elastic constant and the limit of linearity (critical deformation) with respect to the particle concentration is dictated by the fractal nature of the colloidal flocs. Depending on the strength of intra or inter-floc interaction, Shi et al. [43] defined two different regimes: (i) Strong-link regime where inter-floc interactions are stronger than intra-floc (among particles) interactions and (ii) weak-link regime where inter-floc interactions are weaker compared to those among particles (intra-floc). As described in the following equations, in each regime the elastic constant (elastic modulus plateau) and critical deformation describe a scaling relationship with concentration.

(i) Strong-link regime:

(1) $$G_{0}^{\prime }\sim \varphi^{\left(d+x\right)/\left(d-D_{f}\right)}$$

(2) $$\gamma_{0}\sim \varphi^{-\left(1+x\right)/\left(d-D_{f}\right)}$$

where *φ* is the concentration, *d* is the Euclidean dimension of the system (*d* = 3), *D_f_* is the fractal dimension and *x* is the fractal dimension of the backbone that has to be lower than the fractal dimension and larger than the unity, being a reasonable value 1–1.3

(ii) Weak-link regime:

(3) $$G_{0}^{\prime }\sim \varphi^{\left(d-2\right)/\left(d-D_{f}\right)}$$

(4) $$\gamma_{0}\sim \varphi^{1/\left(d-D_{f}\right)}$$

However, these two regimes are two extreme situations. A more realistic situation would be comprehended in between these two behaviors where both inter- and intra-flocs interaction contribute to the elastic constant of the colloidal gel. For that reason, in the Wu and Morbidelli’s model [44], authors introduced a microscopic elastic constant (α) to account for the relative contributions of both inter- and intra-floc links, thus extending the model of Shih et al. as follows:

(5) $$G_{0}^{\prime }\sim \varphi^{\beta /\left(d-D_{f}\right)}$$

(6) $$\gamma_{0}\sim \varphi^{\left(d-\beta -1\right)/\left(d-D_{f}\right)}$$

(7) β=(d−2)+(2+x)(1−α)

where *β* is an auxiliary parameter, *x* is the fractal dimension of the aggregate backbone and usually within the range of 1 to 1.3, and α is the microscopic elastic parameter that comprises the range 0 to 1 (being α = 0 for the strong-link regime and α = 1 for the weak-link regime).

**Figure 4:**
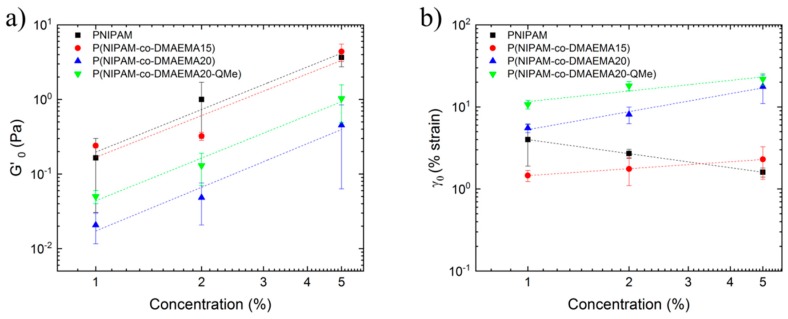
Double-logarithmic plot of (**a**) the elastic modulus plateau, G’_0_, and (**b**) critical strain, *γ*_0_, as a function of the microgel concentration for the samples: PNIPAM, P(NIPAM-*co*-DMAEMA15), P(NIPAM-*co*-DMAEMA20) and quaternized P(NIPAM-*co*-DMAEMA20-QMe).

In order to apply both fractal models, we have collected the elastic modulus plateau, G’_0_, and the critical deformation, *γ*_0_, determined in the strain sweep test (Figure 3), and represented in a double logarithmic plot as a function of microgel concentration (Figure 4). As expected, both G’_0_ and *γ*_0_ exhibited a power law or a scaling relationship with the concentration that can be fitted to the form: G’_0_ ~ C^A^ and *γ*_0_ ~ C^B^ where A and B are the power law exponents (collected in Table 1). In the case of G’_0_ correlation with concentrations, it describes positive slopes (A > 0) in all studied samples. As for the critical deformation, power law exponent for the PNIPAM microgel sample was negative, but with the incorporation of DMAEMA this trend changed to positive values.

According to Shih et al. description of regimes, PNIPAM microgels fall into the strong-link regime, in which inter-floc interactions are stronger than intra-floc: G’ increases with microgel concentration but linear viscoelastic range decreases as *γ*_0_ decreases with concentration. Therefore, the fractal dimension of PNIPAM microgel dispersion could be obtained by applying Equations (1) and (2). As for P(NIPAM-*co*-DMAEMA) microgel samples results, they fall into the weak-link regime (intra-floc interactions predominate in the dispersion), both G’ and *γ*_0_ increased with the concentration of microgels. Therefore, Equations (2)–(4) could be applied to obtain fractal dimension. Both equation systems give also the value of x, backbone fractal dimension. As shown in Table 1, results obtained with the Shih et al. model did not fulfil the requirements mentioned above since negative values of x and *Df* were estimated. Those unrealistic values made the application of the Shih et al. theory not possible for these microgel dispersions.

Having ruled out the Shih et al. model, we applied Wu and Morbidelli’s fractal model (Equations (5)–(7)) so that we could determine fractal dimension (*Df*) and interaction regime (*α*) of the different microgel dispersions (Table 1). The estimation of the interaction regime resulted in values that varied from 0.43 to 0.8, indicating an evolution from a transitioning regime toward a weak-link regime. This means that the incorporation of DMAEMA contributed to the increase in intra-floc interactions, or in other words, DMAEMA contributed to form cluster or agglomerates. This result is also in agreement with the estimation for the fractal dimension *Df* that varied from 1.49 to 2.24 with the incorporation of DMAEMA.

The fractal dimension is the indication of the stacking density of the particles in the floc and also reflects the aggregation mechanism of the dispersion. As established in the literature, there are two growth mechanisms known as reaction-limited and diffusion-limited aggregation [45,46,47]. For the reaction-limited mechanism, the probability for agglomerate collision was low being the agglomeration rate slow. This mechanism ended up in agglomerates with open structures (*Df* ~ 2.0–2.2) [45,46]. In the case of diffusion-limited mechanism, the agglomerate formation was limited by the diffusion and the agglomeration rate was determined by the time between two collisions. In this case, agglomerates formed a closer structure (*Df* ~ 1.7–1.8) [47].

By comparing the fractal dimensions related to each growth mechanism with the obtained fractal dimensions we could conclude that the agglomerate formation for PNIPAM (*Df* ~ 1.43) and P(NIPAM-*co*-DMAEMA15) (*Df* ~ 1.72) microgels followed a diffusion-limited aggregation mechanism. But for the microgel containing higher DMAEMA content, 20% of DMAEMA, aggregates growth followed reaction-limited mechanisms giving rise to a denser packed structure (closer structures) as estimated from the obtained fractal dimension (*Df* ~ 2.24). In the case of methyl quaternized P(NIPAM-*co*-DMAEMA20) the estimated fractal dimension slightly decreased in comparison with the non-quaternized one, which reflected a less dense packed structure. In spite of this, the corresponding aggregates also followed a reaction-limited growth mechanism.

### 3.4. Determination of Microgels Size (Hydrodynamic Diameter) and Surface Charge: Effect of DMAEMA Content and Quaternization

**Figure 5:**
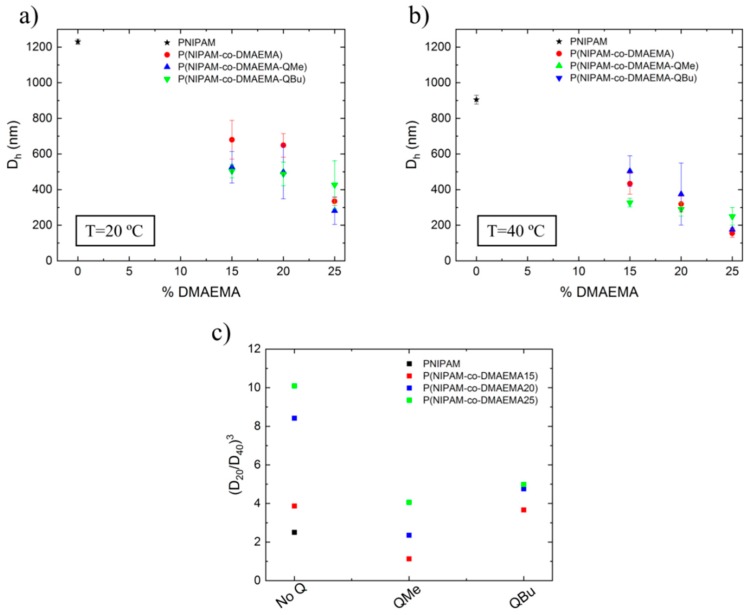
Variation of hydrodynamic diameter of PNIPAM based microgels with the DMAEMA content measured at (**a**) 20 °C and (**b**) 40 °C and (**c**) evolution of the relative swelling index ((D_20_/D_40_^)3^) for unquaternized and quaternized with methyl iodide (QMe) and butyl iodide (QBu) microgels.

Dynamic light scattering measurements were used to determine the hydrodynamic diameter (Dh) of the obtained microgels (Table 2) and to evaluate the influence of the quaternization in the microgel size (Figure 5). Figure 5a,b describes the evolution of microgels Dh with the DMAEMA content, measured at 20 °C and 40 °C, respectively. As seen from both graphs, PNIPAM microgel size decreased substantially with the incorporation of DMAEMA. As stated in the literature, DMAEMA contributed to colloidal stabilization but had a major role during the nucleation step in the precipitation polymerization. Zha et al. [31] observed that the incorporation of DMAEMA favors a short nucleation step, enhances the formation of more precursors and increases the colloidal stability of the nucleated particles that polymerized giving rise to final microgels with smaller size.

If we compare results obtained at 20 °C (Figure 5a) with those obtained at 40 °C (Figure 5b) the hydrodynamic diameter of the samples decreased, as expected for PNIPAM based systems. Therefore, the P(NIPAM-*co*-DMAEMA) crosslinked system still presented thermosensitivity. In this sense, we have determined the relative swelling index of each sample (RSI, (Dh_20_/Dh_40_)^3^) and represented for non-quaternized and methyl/butyl quaternized microgels as shown in Figure 5c. The effect of quaternization either with methyl or butyl iodide derived in a lower (de)swelling index. This could be due to the effect that quaternization might have had in the hydrophilic/hydrophobic balance of the system.

**Figure 6:**
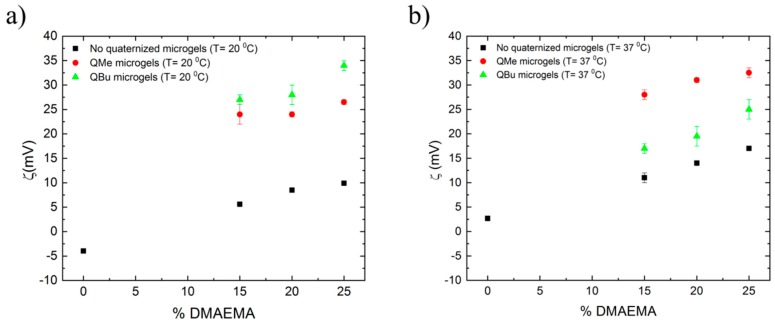
Evolution of zeta potential with DMAEMA content for unquaternized and quaternized microgel samples with (**a**) methyl iodide (QMe) and (**b**) butyl iodide (QBu) alkyl agents, respectively.

We have also determined the effect of DMAEMA content in the surface charge density (zeta potential) of the prepared microgels and analyzed how the quaternization of the tertiary amine of the DMAEMA with different alkyl agents affects the microgel’s surface charge (Figure 6). This characterization was performed at two different temperatures, at 20 °C (Figure 6a) where the microgels were swollen and at 37 °C (Figure 6b) at the collapsed state and physiological condition at which antimicrobial tests were performed as described below. As observed from Figure 6a PNIPAM microgel zeta potential shows a negative value close to the isoelectric point (ISP = 0 mV). When DMAEMA is incorporated to the system, surface charge become positive and increased its value as the content of DMAEMA increased. Interestingly, quaternized microgels, either with methyl or butyl iodide, showed even higher values of zeta potential: 20–25 mV and 27–32 mV, respectively. Similar behavior was observed for zeta potential (ζ) measurements performed at 37 °C. As seen in Figure 6b, PNIPAM microgel shows a not very significant increase going from negative to positive zeta potential values when increasing temperature, however, both values were close to the isoelectric point. In the case of quaternized microgels, substantial differences were observed depending on the alkyl agent used for the quaternization. In the case of QMe microgels, no important differences were observed with temperature, being the obtained zeta potential at 37 °C, vaguely higher than that measured at 20 °C. This was not the case for QBu microgels which showed a significant decrease of ζ when compared to the values obtained at 20 °C. This result could have been due to the thermo-responsiveness of PNIPAM, in particular to the coil-to-globule transition that occurs in response to a hydrophilic-to-hydrophobic character change upon heating. It is possible to think that butyl chain incorporated to the tertiary amine in the quaternization reaction could somehow mask the positive charges of the surface when microgel was in its collapsed state, so that microgel surface charge became less positive.

### 3.5. Evaluation of Unquaternized and Quaternized (QMe, QBu) Microgels Antimicrobial Activity

We have evaluated the antimicrobial activity of all prepared microgels against typical microorganisms causing hospital related infection diseases. The analysis of antimicrobial activity was studied in terms of minimum inhibitory concentration (MIC) against gram-positive bacteria strains (*S. aureus* and *S. epidermidis)* and a fungus (*C. parapsilosis*) estimated by standard broth microdilution methods. Gram-negative *Eschericia coli* and *Pseudomona aeruginosa* bacteria were also tested but no activity was obtained at the concentrations tested. The measurements were done in triplicate and the results are summarized in Table 3.

As observed from the results, PNIPAM microgel does not exhibit any activity as expected. Interestingly, the incorporation of DMAEMA up to 25 wt% into the microgel structures provided some biocidal properties, although the MIC values were still high. Previous studies have also shown that DMAEMA homopolymer exhibits slight antimicrobial activity without quaternization [48] and in this case we can see that DMAEMA units gave to microgel, certain antimicrobial properties. The quaternization with methyl iodide, however, does not improve much the biocidal activity against either bacteria or fungi, although promising results were found for the microgel with higher content of DMAEMA, with a MIC value of 2.5 mg mL^−1^ against *S. epidermidis* bacteria. In contrast the samples quaternized with butyl iodide showed no killing activity. As stated in previous work, the antimicrobial activity might decrease with the alkyl length, as the hydrophobicity of the system increases, fact that limits the accessibility of the positive charges [33,49]. Therefore, as a possible approach to improve the effectiveness of these microgels, (which are not very effective at this point), is to increment the DMAEMA content without compromising their stability and responsiveness properties. The results found in the microbiological test were correlated with the results obtained in D_h_ and zeta potential measurements, where important differences were detected between QMe and QBu microgel samples. The samples quaternized with methyl iodide presented the highest ζ values, thus the highest available positive charges, and so better antimicrobial activity was expected. The microgels quaternized with butyl iodide exhibited low positive zeta potential values at 37 °C (Figure 6b), low D_h_, and a relative swelling index; facts that demerit their interaction with the microorganism and, thereby, their actions against them.

Therefore, it was demonstrated that the incorporation of DMAEMA units into the NIPAM microgel structure, in particular those quaternized with methyl iodide imparted certain antimicrobial character. In addition, it was shown that the microgels maintained the thermo-responsive behavior with the incorporation of DMAEMA; so these microgels can be promising systems for the preparations of antimicrobial materials with dual action, with inherent antimicrobial activity by contact and on second hand, as controlled release systems of antimicrobial agents.

## 4. Conclusions

In this work P(NIPAM-*co*-DMAEMA) microgels have been successfully synthesized and further quaternized via N-alkylation reaction using as alkyl agent methyl iodide and butyl iodide. DLS measurements, performed to evaluate the effect of DMAEMA incorporation and its quaternization, confirmed that (i) the incorporation of DMAEMA ended up in a decrease of the hydrodynamic diameter, as expected, and (ii) that the incorporation of up to 25 wt.% of DMAEMA did not eliminate temperature responsiveness of PNIPAM, as observed from the analysis of Dh at 20 °C and 40 °C, swollen and collapsed state of the microgels, respectively. From zeta potential measurements we have confirmed that the incorporation of DMAEMA contributed to colloidal stability, increasing the ζ towards positive values. This value was further increased when tertiary amine was quaternized. However, temperature had an opposite effect in the surface charge of the quaternized microgels, being the one quaternized with methyl iodide the most positive and stable in terms of colloidal stability.

Considering potential applications as drug delivery systems, we believed it crucial to understand its rheological behavior; namely, how it could respond under deformation. For that, two scaling models were applied. Interestingly, significant influence of DMAEMA in the regime of interactions was detected. Increased DMAEMA content induced microgel dispersions to evolve from a strong-link toward a weak-link regime where interactions among particles dominated the elastic constant of the system.

Since the final purpose of the work was the development of intrinsically antimicrobial microgels, a test regarding antimicrobial activity was also performed. As it could be expected from ζ measurements, methyl quaternized microgels with 25% wt. of DMAEMA showed the lowest MIC values. Although the obtained results are far from be strongly effective, we believe that there is still room for improvement. In addition, these microgels, able to respond to several external stimuli, can also be loaded with different drugs so that a tailored multifunctional antimicrobial system could be developed.

## Figures and Tables

**Table 1:** Summary of the results obtained by applying Shi et al., and Wu and Morbidelli’s fractal models.

Samples	Slopes from Figure 4		Shih et al. Model		Wu and Morbidelli Model		
	A	B	*Df*	x	*Df*	α	Regime
PNIPAM	1.89	−0.56	1.49	<0	1.49	0.43	Transition (strong)
P(NIPAM-*co*-DMAEMA15)	1.85	0.28	<0	---	1.72	0.58	Transition (weak)
P(NIPAM-*co*-DMAEMA20)	1.94	0.72	1.16	<0	2.24	0.85	Weak-link
P(NIPAM-*co*-DMAEMA20-QMe)	1.89	0.42	0.6	1.5	2.13	0.80	Weak-link

**Table 2:** Hydrodynamic diameter (Dh) of the studied microgels measured at 20 °C and 40 °C. Determination of the relative swelling index (RSI).

Samples	Dh_20_ (nm) (20 °C)	Dh_40_ (nm) (40 °C)	RSI (D_20_/D_40_)^3^
PNIPAM	1230 ± 14	905 ± 25	2.51
P(NIPAM-*co*-DMAEMA15)	680 ± 109	433 ± 58	3.87
P(NIPAM-*co*-DMAEMA15-QMe)	526 ± 89	504 ± 86	1.14
P(NIPAM-*co*-DMAEMA15-QBu)	504 ± 37	327 ± 24	3.66
P(NIPAM-*co*-DMAEMA20)	649 ± 66	319 ± 44	8.42
P(NIPAM-*co*-DMAEMA20-QMe)	499 ± 151	375 ± 174	2.35
P(NIPAM-*co*-DMAEMA20-QBu)	488 ± 66	290 ± 38	4.76
P(NIPAM-*co*-DMAEMA25)	355 ± 24	155 ± 23	10.10
P(NIPAM-*co*-DMAEMA25-QMe)	281 ± 76	176 ± 9	4.06
P(NIPAM-*co*-DMAEMA25-QBu)	427 ± 135	250 ± 49	4.98

**Table 3:** Minimum inhibitory concentration (MIC) of microgels against gram-positive bacteria and fungi.

Samples	MIC (mg/mL)		
	*S. aureus*	*S. epidermidis*	*C. parapsilosis*
PNIPAM	>10	>10	>10
P(NIPAM-*co*-DMAEMA15)	5	5	5
P(NIPAM-*co*-DMAEMA20)	5	5	5
P(NIPAM-*co*-DMAEMA25)	5	5	5
P(NIPAM-*co*-DMAEMA15-QMe)	5	5	5
P(NIPAM-*co*-DMAEMA20-QMe)	5	5	5
P(NIPAM-*co*-DMAEMA25-QMe)	5	2.5	5
P(NIPAM-*co*-DMAEMA15-QBu)	>10	>10	>10
P(NIPAM-*co*-DMAEMA20-QBu)	>10	>10	>10
P(NIPAM-*co*-DMAEMA25-QBu)	>10	>10	>10

## Supplementary Materials

The following are available online at https://www.mdpi.com/2073-4360/11/4/606/s1, Figure S1: (a) ^1^H-NMR spectrum corresponding to P(NIPAM-*co*-DMAEMA25), P(NIPAM-*co*-DMAEMA25-QMe) and P(NIPAM-*co*-DMAEMA25-QBu). (b) P(NIPAM-*co*-DMAEMA) microgel quaternization reaction; Figure S2: DSC thermorgrams during second heating scan of (a) P(NIPAM-*co*-DMAEMA) (b) P(NIPAM-*co*-DMAEMA-QMe) and P(NIPAM-*co*-DMAEMA-QBu).
