# Supplementary material for: Thermoresponsive Poly(N-Isopropylacrylamide-co-Dimethylaminoethyl Methacrylate) Microgel Aqueous Dispersions with Potential Antimicrobial Properties
Source: Polymers (Basel). 2019 Apr 2;11(4):606. doi: 10.3390/polym11040606 (PMC6523738; doi:10.3390/polym11040606)
Supplement: Supplementary file 1 [file polymers-11-00606-s001.pdf]

SUPPORTING INFORMATION

Thermoresponsive Poly(N-Isopropylacrylamide-*co*-Dimethylaminoethyl Methacrylate) Microgel Aqueous Dispersions With Potential Antimicrobial Properties

Coro Echeverría[1,2]*, Alejandro Aragón-Gutiérrez[1], Marta Fernández-García[1,2], Alexandra Muñoz-Bonilla[1,2], Daniel López[1,2]*

[1]Instituto de Ciencia y Tecnología de Polímeros (ICTP-CSIC), C/Juan de la Cierva 3, 28006 Madrid, Spain.

[2]Interdisciplinary Platform for Sustainable Plastics towards a Circular Economy-Spanish National Research Council (SusPlast-CSIC), Madrid, Spain.

* Correspondence: cecheverria@ictp.csic.es; daniel.l.g@csic.es

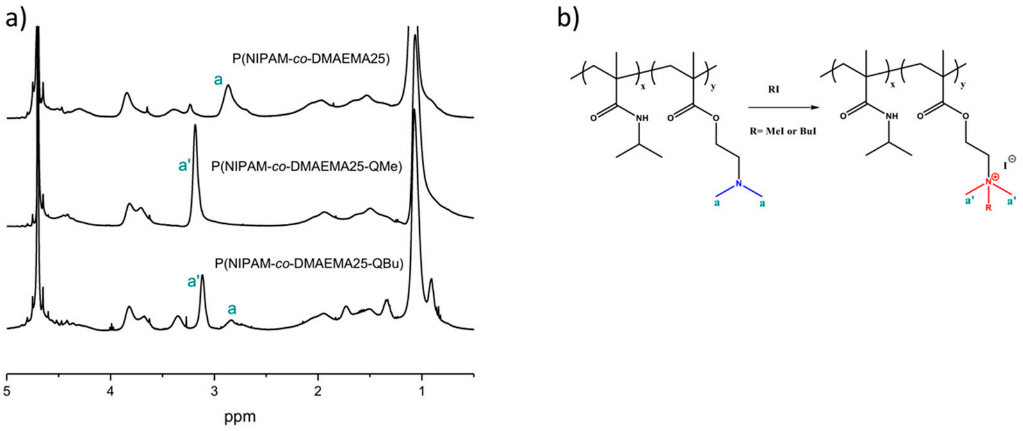

Figure S1. a) ^{1}H-NMR spectrum corresponding to P(NIPAM-*co*-DMAEMA25), P(NIPAM-*co*-DMAEMA25-QMe) and P(NIPAM-*co*-DMAEMA25-QBu). b) P(NIPAM-*co*-DMAEMA) microgel quaternization reaction.

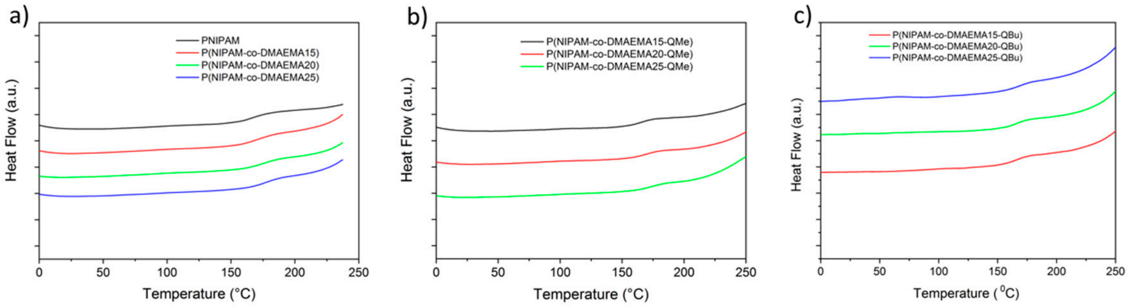

Figure S2. DSC thermograms during second heating scan of a) P(NIPAM-*co*-DMAEMA); b) P(NIPAM-*co*-DMAEMA-QMe) and P(NIPAM-*co*-DMAEMA-QBu).
